# Monitoring the Epidemiology of Otitis Using Free-Text Pediatric Medical Notes: A Deep Learning Approach

**DOI:** 10.3390/jpm14010028

**Published:** 2023-12-25

**Authors:** Corrado Lanera, Giulia Lorenzoni, Elisa Barbieri, Gianluca Piras, Arjun Magge, Davy Weissenbacher, Daniele Donà, Luigi Cantarutti, Graciela Gonzalez-Hernandez, Carlo Giaquinto, Dario Gregori

**Affiliations:** 1 Unit of Biostatistics, Epidemiology and Public Health, Department of Cardiac, Thoracic, Vascular Sciences and Public Health, University of Padova, 35131 Padova, Italy; corrado.lanera@unipd.it (C.L.); giulia.lorenzoni@unipd.it (G.L.);; 2 Division of Pediatric Infectious Diseases, Department for Woman and Child Health, University of Padova, 35128 Padova, Italy; elisa.barbieri@unipd.it (E.B.); daniele.dona@unipd.it (D.D.); carlo.giaquinto@unipd.it (C.G.); 3 Health Language Processing Center, Institute for Biomedical Informatics at the Perelman School of Medicine, University of Pennsylvania, Philadelphia, PA 19104, USA; amaggera@asu.edu (A.M.); dweissen@pennmedicine.upenn.edu (D.W.); gragon@pennmedicine.upenn.edu (G.G.-H.); 4 Società Servizi Telematici—Pedianet, 35100 Padova, Italy; l.cantarutti@sosepe.com

**Keywords:** otitis, machine learning, text mining, electronic medical record, real-world data

## Abstract

Free-text information represents a valuable resource for epidemiological surveillance. Its unstructured nature, however, presents significant challenges in the extraction of meaningful information. This study presents a deep learning model for classifying otitis using pediatric medical records. We analyzed the Pedianet database, which includes data from January 2004 to August 2017. The model categorizes narratives from clinical record diagnoses into six types: no otitis, non-media otitis, non-acute otitis media (OM), acute OM (AOM), AOM with perforation, and recurrent AOM. Utilizing deep learning architectures, including an ensemble model, this study addressed the challenges associated with the manual classification of extensive narrative data. The performance of the model was evaluated according to a gold standard classification made by three expert clinicians. The ensemble model achieved values of 97.03, 93.97, 96.59, and 95.48 for balanced precision, balanced recall, accuracy, and balanced F1 measure, respectively. These results underscore the efficacy of using automated systems for medical diagnoses, especially in pediatric care. Our findings demonstrate the potential of deep learning in interpreting complex medical records, enhancing epidemiological surveillance and research. This approach offers significant improvements in handling large-scale medical data, ensuring accuracy and minimizing human error. The methodology is adaptable to other medical contexts, promising a new horizon in healthcare analytics.

## 1. Introduction

Otitis media (OM) is one of the most prevalent infectious diseases in childhood, causing high healthcare resource utilization, especially in primary care settings [1]. Moreover, the indiscriminate use of antibiotics for the treatment of OM has been recorded, contributing to an increase in the burden of antibiotic resistance [2]. In recent years, it has been suggested that introducing pneumococcal conjugate vaccines has resulted in a change in OM epidemiology and antibiotic prescriptions [3]. Vaccine administration seems to result in a reduction in pneumococcal Acute OM (AOM); however, the efficacy on all-cause AOM is still debated, so OM continues to represent a severe burden from a public health perspective, justifying the need for the strict epidemiological surveillance of the phenomenon [4,5]. The regular monitoring of OM enables the identification of patterns and trends in incidence, informing prevention strategies and healthcare policies. Moreover, it aids in understanding the etiology and risk factors associated with otitis, which is essential for developing targeted interventions. Such surveillance also facilitates the early detection of emerging resistant pathogens, guiding appropriate antibiotic use. Ultimately, the effective surveillance of pediatric otitis supports public health initiatives, reduces healthcare burdens, and contributes to the overall well-being of children.

Unfortunately, studying the epidemiology of OM can be challenging because this type of disease is treated mainly in an outpatient setting, resulting in difficulties in accessing data collected by pediatricians. In Italy, the main challenge is represented by using the International Classification of Disease (ICD) codes for classifying patients’ diagnoses, which is not mandatory for pediatricians working in an outpatient setting. Not least, using narratives to report data collected (e.g., for reporting clinical notes) is still common [6].

In this context, narratives can be considered a valuable source of information. In a recent study, integrating free-text clinical notes with structured information enhanced the diagnostic accuracy of acute respiratory infections encountered during outpatient visits [7]. It is worth noting that exploiting narratives can be demanding, both in terms of labor resources and costs. An automatic machine learning approach to the problem is appealing, given machine learning’s potential to effectively use all the available textual information to detect the information reported in narratives. Furthermore, a deep learning approach can effectively take advantage of data that are orders of magnitude larger than classical shallow machine learning models [8].

The application of automatic free-text classification in the field of pediatric infection epidemiology presents substantial opportunities for advancement. Utilizing deep learning for the diagnosis and monitoring of otitis in children has the potential to revolutionize data collection and analysis in epidemiological studies. Firstly, deep learning enables the efficient processing of large volumes of unstructured data, such as clinical notes and medical reports, which is particularly advantageous in areas with extensive and complex datasets [9]. Secondly, automatic classification enhances accuracy and consistency in case categorization. Traditionally, this task requires significant human effort, making it prone to errors and variability. Deploying deep learning models reduces the risk of misclassification and ensures greater uniformity in data interpretation. Thirdly, automation allows for the real-time monitoring of epidemiological trends. This is crucial for the prompt identification of outbreaks or shifts in infection patterns, enabling timely and targeted interventions [10]. Additionally, the ability to analyze free text paves the way for utilizing unconventional data sources like social media and online platforms, which can provide supplementary insights into infection spread dynamics. Finally, deep learning models can aid in personalizing care. By analyzing detailed clinical narratives, it is possible to identify patterns specific to patient groups, contributing to more targeted and effective medicine for pediatric infections. This approach is particularly valuable in contexts where individual variations significantly influence infection outcomes, as in the case of otitis in children.

The present work proposes an automatic deep learning system trained using data from Pedianet to classify otitis from outpatient clinical records into six mutually exclusive categories: no otitis, otitis (not media or acute), OM (not acute), AOM, AOM with perforation, or recurrent AOM (when explicitly stated by the pediatrician in the electronic health record).

## 2. Materials and Methods

The data used for the present work came from the Pedianet database, which contains information on 6,903,035 visits of 216,976 children (collected by 144 family pediatricians from 1 January 2004 to 23 August 2017). Pedianet [11] is an Italian pediatric general practice research database. It contains information on the reason for the visit, health status (according to the Guidelines of Health Supervision of the American Academy of Pediatrics), personal details, growth parameters, diagnosis, and clinical details (free text or the ICD-9), prescriptions (pharmaceutical prescriptions identified by the Anatomical-Therapeutical Chemical code, specialist appointments, diagnostic procedures, hospital admissions), and outcome data for the children. Pediatricians can access the system using standard software (JuniorBit®, Version 7.2.7) during routine patient care, and then, data are anonymized and sent monthly to a centralized database in Padova for validation. The data used in the present study for the classification task included textual information.

Records relevant to the classification were identified from the main database through a search string similar to the one used by Barbieri et al. [2] but looking at all the free-text fields. The string was built to include various potential typographical errors and abbreviations (Table S1, Supplementary Materials).

We split the records in the main database into three main sets: the training, validation, and test sets. Each dataset was obtained through a sampling strategy that ensured the same proportion of patients per pediatrician was maintained as in the main database; at least 500 records were included in each dataset, and at least one record for each pediatrician was included. The training set was derived from historical records (2004–2007), while the validation and test sets were derived from records reported in the main database after 2007.

### 2.1. Gold Standard

The classification of the otitis cases (gold standard) was performed by two independent evaluators (experts in pediatric care). The otitis cases were classified according to six mutually exclusive classes: no otitis, otitis (not acute), OM (not acute), AOM (without tympanic membrane perforation or recurrence), AOM with tympanic membrane perforation, and—recurrent AOM. For recurrent AOM cases, the definition used was the one of Goycoolea: “the condition in a child is defined as having at least three episodes of AOM in a period of six months, or four or more episodes in 12 months”, or with an explicit statement of the pediatricians which mark the case as recurrent [12].

Disagreements between the two independent evaluators were solved by a third independent reviewer who was specialized in infectious diseases. The agreement between the reviewers was then evaluated using the weighted Cohen’s kappa.

### 2.2. Data Pre-Processing

To process the free text in our study, we employed the fastText algorithm [13] with a skip-gram architecture on our main database, including various medical texts, to create dense word vectors in a 300-dimensional space. This resulted in a dictionary linking each word to a 300-dimensional vector, totaling 122,591 entries.

For data preparation, we merged all text fields from each medical visit into a single text stream using “SEP” as a separator between fields and replacing all numbers with “NUM” to simplify the dataset. Our embedding dictionary included an “OOV” token for future out-of-vocabulary words, represented by a small random vector.

Each network input was a 2-dimensional tensor, with dimensions representing batch size and a fixed word count per record (1000 words), using padding or truncation as needed. Based on our embedding dictionary, the first network layer transformed this input into the proper tensor.

### 2.3. Model Development

In the initial training stage, we utilized the training dataset to identify the optimal set of parameters for each model architecture. Subsequently, the most effective model from each architecture was retrained, incorporating both the original training set and a randomly selected subset of the validation set comprising 300 records. Following this, the validation set was employed to fine-tune these trained networks. This involved assessing their performance with various hyperparameter configurations to determine the most effective combination. The ultimate selection of the best models, along with their combined ensemble, was then evaluated on the test set. The performance was evaluated by computing the accuracy, balanced precision, balanced recall, and balanced F1 measure.

### 2.4. Architectures Employed for Model Development

Several deep learning architectures were explored and tuned on the validation set, and an ensemble model was constructed based on them [14]. The Adam optimizer trained all the networks to minimize the average training cross-entropy loss function among the batches [15].

We explored different architectures. Common parts of all of them were provided as inputs, the first hidden layer (i.e., the embedding) and the output layer. The output layer had six neurons to represent all the possible classes. It was activated by the logit function and processed by the softmax function.

We applied batch normalization after each hidden layer to maintain the control of both exploding and vanishing gradient events [16]. To avoid overfitting, we considered a dropout layer, i.e., a layer that randomly ignored a random set of neurons given a rate after each hidden layer once batch-normalized. For the embedding layer, we considered a dropout ratio of 0.2, while for the others, we explored two ratios: 0.5 and 0.7 [17]. With regard to the batch size, for each network, we explored two options: M = 8 or M = 16 [18].

The architecture explored was the following benchmark architecture (i.e., a simple embedding: “0”) plus four others of increasing complexity:

0. Simple embedding: The only hidden layer was the embedding layer.
1. Single kernel convolutional neural network (CNN): After the embedding layer, we attached a single convolutional layer.
2. Sequential single kernel CNN: After the embedding layer, we attached a sequence of two convolutional layers.
3. Multiple parallel kernel CNN: After the embedding layer, we attached a single concatenation of multiple convolutional layers.
4. Deep multiple parallel kernel CNN: After the embedding layer, we attached a sequence of two distinct concatenations of multiple convolutional layers.

The final ensemble model comprised the four networks described (simple embedding excluded), considering the mean of all their probability predictions for each class estimated by their output layer before applying their softmax activation function. The same softmax was subsequently applied to determine the class assigned to the record by the final ensemble.

We ran all the computations on an Ubuntu 18.04.3 LTS GNU/Linux 4.15.0-58-generic x86_64 virtualized server in the Unit of Biostatistics, Epidemiology, and Public Health at the University of Padova, equipped with 16 cores from Intel^®^ Xeon^®^ CPUs E5-2640 v4 @ 2.40 GHz, and 96 GiB-RAM. We implemented all the networks and codes for the analyses in R (v3.6.1), powered by the Keras (v2.2.4.1.9001) R interface to the TensorFlow (v1.14) backend, built from source code enabling the usage of the Intel^®^ AVX set of instruction extensions. To learn word representation, fastText (v0.9.1) was used. Diagrams for the networks were automatically drawn from the Keras models trained using Netron (v3.3.5). All the developments and code were tracked on a GitHub repository that is available to the public at www.github.com/UBESP-DCTV/limpido (accessed on 22 December 2023).

## 3. Results

**Figure 1:**
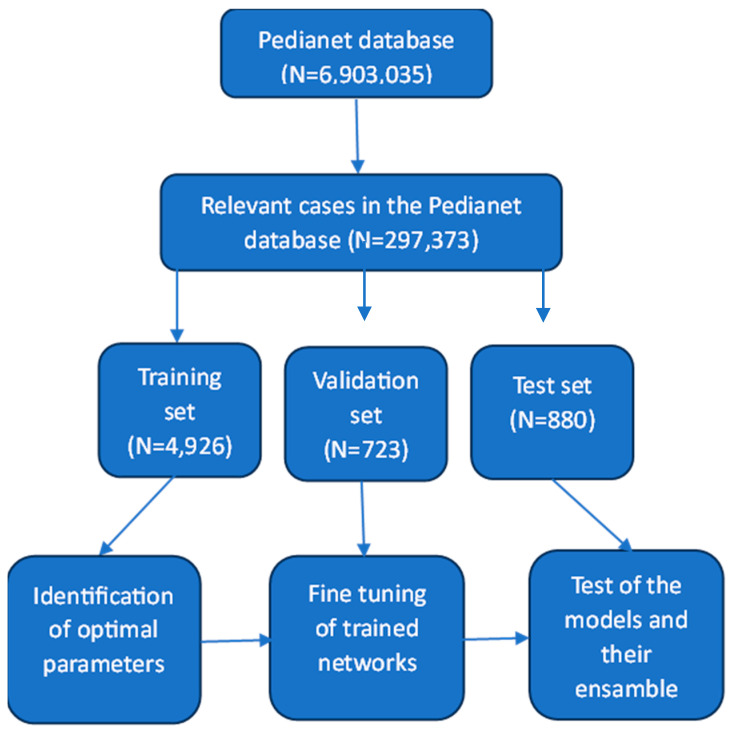
Study workflow.

Figure 1 presents this study’s workflow.

The original Pedianet dataset included 6,903,035 records corresponding to 216,976 children. After identifying the relevant cases, the main dataset comprised 297,373 records corresponding to 99,896 children and 142 pediatricians. The children in the main database were slightly more likely to be male (52.2%).

The training set (Table 1) included 4926 records corresponding to 4475 children. The validation set included 723 visits corresponding to 718 children, and the test set included 880 visits corresponding to 873 children. The proportion of children of the male gender was constant among the three datasets and in line with that in the main dataset (~52%).

**Figure 2:**
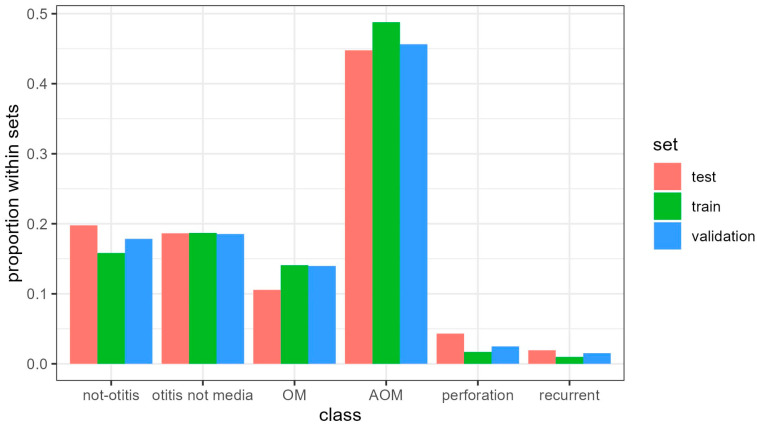
Proportion of diagnostic classes according to the gold standard classification in the three datasets used for model development.

Figure 2 presents the distribution of diagnostic classes according to the gold standard classification in the three datasets. Cases of AOM without perforation represented the highest proportion in all three datasets, ranging from 44% to 48%. The agreement between the independent reviewers was very good, with a weighted Cohen’s Kappa value of 0.89.

### Model Performance

Tables S2–S5 (Supplementary Materials) report the performances on the validation set for the models 1–4. The validation performance was excellent, with accuracy values of at least 0.9. Table 2 presents the confusion matrix for the final ensemble model in the test set. Thirty were the cases misclassified by the ensemble model. One of the most common errors included no otitis being incorrectly predicted as otitis. A detailed review by a pediatrician of the 30 records incorrectly classified by the ensemble model revealed that these misclassifications often occurred due to negations related to otitis (four instances) or references to other doctors’ diagnoses, predispositions, or uncertain cases. Similarly, perforation was frequently misclassified as AOM, which was observed in six cases where doctors described eardrum perforation using atypical terminology.

The performances on the test set for the best model selected and their ensembles are reported in Table 3. The performance of the single models was generally very good. The accuracy ranged from 81.70 to 96.59. Similarly, the balanced precision ranged from 84.51 to 96.95, and the balanced F1 measure ranged from 75.75 to 95.86. Taking the performance metrics together, the ensemble model outperformed the other architectures, with values of 97.03, 93.97, 96.59, and 95.48 for balanced precision, balanced recall, accuracy, and balanced F1 measure, respectively.

## 4. Discussion

In this work, we considered a deep learning approach for a multiclass classification problem. In particular, we used the Pedianet database as a source of information to classify free-text diagnoses reporting no otitis, otitis (not media or acute), OM (not acute), AOM, AOM with perforation, or recurrent AOM. We trained models using five different deep learning architectures, from which a final ensemble model was developed.

Free-text information represents a valuable resource for epidemiological surveillance. Its unstructured nature, however, presents significant challenges in the extraction and interpretation of meaningful information since the manual classification of this type of information is often time- and resource-consuming, requiring extensive human effort and expertise, which can lead to inconsistencies and errors in large datasets. The advent of deep learning techniques has revolutionized this landscape. These techniques, characterized by the ability to learn hierarchical representations, have shown remarkable progresses in exploiting free text. These approaches offer several advantages, such as reducing the labor dedicated to diagnosis and real-time classification. However, this is only true if these automatic systems reach performances comparable to human levels [19,20].

The present study’s results highlight the feasibility of deep learning models for automatically classifying free-text information reported in medical records, in line with previous research in pediatrics. A machine learning approach was adopted for the automatic extraction of free-text diagnoses reported in the emergency department records in Nicaragua with a median accuracy of almost 80% [21]. Another study on the Pedianet dataset employed a set of machine learning techniques (GLMNet, multinomial logistic regression (MAXENT), and the boosting approach LogitBoost) for predicting varicella cases. The performance on the test set showed that the highest predictive values were reached with the boosting algorithm (positive predictive value 63.1, 95% CI 42.7–83.5, and negative predicative value 98.8, 95% CI 98.3–99.3) [22]. In another study using the same Pedianet database, deep learning approaches were demonstrated to be feasible for detecting varicella cases for epidemiological surveillance, showing an area under the ROC curve (AUC-ROC) of 95.30% [23]. The results of this study are also consistent with a recent study on diagnoses evaluation for pediatric diseases from EHRs by Liang et al. [24] on more than one hundred million EHRs of over a million children, with excellent F1 values.

Deep learning models represent a valuable opportunity for free text exploitation since they can take advantage of datasets that are orders of magnitude larger than classical machine learning models [8]. Another advantage of deep learning approaches, particularly relevant in the context of pediatric otitis surveillance, is their ability to improve iteratively over time. This process involves starting from a model that has already undergone initial training rather than building a model from scratch for each new task. This method not only saves significant time and resources but also allows for the leveraging of the pre-existing knowledge embedded within the model. Such an approach is especially useful when considering reduced models, where some of the last layers might be excluded. By doing so, a substantial portion of the knowledge from a model that has already demonstrated high performance on a specific task can be repurposed. This existing knowledge base serves as an invaluable starting point for training a new model on a different yet related task [25]. For example, a model trained to identify patterns in general pediatric diseases can be fine-tuned to specifically recognize the nuances of otitis in children. This capability to transfer and adapt learned patterns across different but similar tasks is a hallmark of deep learning’s flexibility and efficiency. It enables the development of more specialized models that can address unique challenges in pediatric epidemiology, leading to more accurate diagnoses and better-informed healthcare strategies. This means, on the one hand, that our model could possibly be useful as a basis for training other deep learning models to classify different infections. Furthermore, considering that deep learning models can be merged to combine their knowledge, our results and methodology may also be of interest for improving other deep learning models. By integrating our model with others, the collective intelligence can address more complex or rare infections, offering a robust, comprehensive tool for medical professionals. This collaborative aspect underscores the transformative potential of deep learning in healthcare.

Recently, the advent of Generative Pre-trained Transformer (GPT) models has opened new possibilities in natural language processing, particularly in biomedical applications [26]. An increasing number of studies have presented a variety of applications using GPT models in biomedical contexts [27], with initial studies showing promising results [28,29]. GPT models offer distinct advantages over in-house-developed models; for example, they eliminate the need for text pre-processing, training, and managing computational aspects through cloud-based solutions. However, using third-party models such as GPT models raises significant bioethical [30] and privacy concerns, particularly regarding the sharing of sensitive data. In addition, the development of in-house natural language processing models allows researchers to have greater control over algorithm development and functionality, ensuring a customized approach to specific research needs. This is critical in the medical field, where accuracy and the ability to understand and modify the mechanisms underlying the model can have significant implications for both the reliability of results and patient outcomes. GPT models, while powerful in processing and generating text, often lack transparency in their operational mechanisms and decision-making processes. One major issue is the difficulty in understanding how GPT models arrive at specific outputs. In a clinical setting, it is crucial to trace the reasoning behind a diagnosis or treatment recommendation. This lack of interpretability can lead to skepticism and resistance from healthcare professionals, who are accustomed to evidence-based practices and clear, explainable decision pathways.

### Exploiting Free-Text Information in Biomedical Research: Practical Implications

In the biomedical domain, the integration of underutilized free-text data with structured information holds significant potential for advancing research and clinical care [31]. Traditionally, biomedical research has heavily relied on structured data due to its ease of quantification and analysis. However, this approach often overlooks the rich, nuanced information contained in free text, such as doctors’ notes, patient journals, and clinical narratives, which can provide invaluable insights into patient care and disease progression. One primary advantage of integrating these data types is the enrichment of the data pool with qualitative insights. Free-text sources frequently contain subjective patient experiences, specific symptom descriptions, and detailed treatment responses, all of which are typically absent in structured datasets. By incorporating these elements, researchers can achieve a more holistic understanding of medical conditions, leading to better-informed hypotheses and more comprehensive study designs. Additionally, free-text data can serve as a crucial tool for hypothesis generation and validation. It can reveal previously unrecognized correlations or patterns, prompting new research inquiries and supporting existing theories. For example, the detailed descriptions of patient responses to treatments in clinical notes can inform more effective therapeutic strategies. Furthermore, integrating free text with structured data can significantly enhance the accuracy of machine learning models used in biomedical research. The nuances and depth of information in free text can complement the structured data, providing a richer dataset for algorithm training and improving the predictive power of these models. In summary, leveraging often underutilized free-text data in conjunction with structured information can help to uncover new dimensions of medical research, enhance the depth and quality of analyses, and, ultimately, contribute to more effective and personalized patient care in the biomedical field.

## 5. Conclusions

Our analysis confirmed the potential of deep learning models in identifying and classifying diagnoses from free text. These methodologies could be adopted in other healthcare databases to improve healthcare research and limit human errors and time-speeding database interrogations.

Monitoring for otitis incidence and prevalence from pediatricians’ diaries can be automated with accuracy and timeliness via deep learning models. This approach significantly enhances the efficiency and reliability of data collection in pediatric epidemiology. By harnessing the power of deep learning, the model can swiftly process vast amounts of text data, extracting relevant information with a high degree of precision. This capability ensures that the surveillance of otitis is not only faster but also more accurate, enabling healthcare professionals to identify trends and outbreaks promptly. Such timely and accurate monitoring is vital for implementing effective public health interventions and improving outcomes for pediatric patients.

## Figures and Tables

**Table 1:** Characteristics (number of visits, number of children, number of pediatricians, gender of children) of the training, validation, and test sets used for model development and testing. For categorical variables, data are absolute numbers (percentages).

	Training Set	Validation Set	Test Set
Visits	4926	723	880
Pediatricians	138	142	142
Children	4475	718	873
Gender: Male	2349 (52.5%)	377 (52.5%)	463 (53.0%)
Females	2078 (46.4%)	341 (47.5%)	410 (47.0%)

**Table 2:** Confusion matrix for the classes predicted by the ensemble model (row) and according to the gold standard (columns).

Predicted\Gold	No Otitis	Otitis Not Media	OM	AOM	Perforation	Recurrent	Sum
No otitis	155	0	2	0	0	0	157
Otitis not media	7	168	7	0	0	0	182
OM	1	0	101	1	1	0	104
AOM	2	1	1	389	6	0	399
Perforation	0	0	0	1	28	0	29
Recurrent	0	0	0	0	0	9	9
Sum	165	169	111	391	35	9	880

**Table 3:** Performances on the test set evaluated on the best model of each architecture and on the ensemble model.

Selected Network	Balanced Precision	Balanced Recall	Accuracy	Balanced F1
Simple embedding	84.51	68.63	81.70	75.75
Single kernel	92.60	91.87	94.66	92.23
Sequential CNN	95.94	81.26	93.64	87.99
Parallel CNN	96.95	94.78	96.59	95.86
Deep CNN	96.38	93.36	96.25	94.85
Ensemble (w/o simple embeddings)	97.03	93.97	96.59	95.48

## Data Availability

The data analyzed in this study are subject to the following licenses/restrictions: The data used in this study cannot be made available in the article, the Supplementary Materials, or in a public repository due to Italian data protection laws. The anonymized datasets generated during and/or analyzed during the current study will be provided upon reasonable request to the corresponding author after written approval is granted by the Internal Scientific Committee. Requests to access these datasets should be directed to the Internal Scientific Committee (info@pedianet.it).

## Supplementary Materials

The following supporting information can be downloaded at: https://www.mdpi.com/article/10.3390/jpm14010028/s1, Table S1: Regular expressions used to filter possible cases of otitis from the Pedianet databases. The final regular expression applied was the disjunction of the reported expressions (i.e., linked with the “OR” Boolean operator). Table S2: Performances for the single kernel CNN architectures. Table S3: Performances for the sequential kernel CNN architectures. Table S4: Performances for the parallel kernel CNN architectures. Table S5: Performances for the deep-parallel kernel CNN architectures.
