# Supplementary material for: Monitoring the Epidemiology of Otitis Using Free-Text Pediatric Medical Notes: A Deep Learning Approach
Source: J Pers Med. 2023 Dec 25;14(1):28. doi: 10.3390/jpm14010028 (PMC10817419; doi:10.3390/jpm14010028)
Supplement: Supplementary file 1 [file jpm-14-00028-s001.zip › jpm-2775829-supplementary.pdf]

Supplementary Material

Table S1. Regular expressions used to filter possible cases of otitis from the Pedianet databases. The final regular expression applied was the disjunction of the reported expressions (i.e., linked with the “OR” Boolean operator)

<table>
<tr><th>Regular expression</th></tr>
<tr><td>"o{1,2}tit|ot{1,2}it|oti{1,2}t|otit{1,2}|toite|oitte|otiet|^om|^o\\.m|\\som|\\so\\.m\\.|\\Wom|\\Wo\\.m\\.|\\dom|\\do\\.m\\.|\\nom|\\no\\.m\\."</td></tr>
<tr><td>"\\so\\stit|ot\\sit|oti\\st|\\so\\m\\s|\\so\\.\\sm\\.\\s"</td></tr>
<tr><td>"\\sitite|\\s9tite|\\s0tite|\\sptite|\\sltite|\\sktite"</td></tr>
<tr><td>"\\sorite|\\so5ite|\\so6ite|\\soyite|\\sogite|\\sofite"</td></tr>
<tr><td>"\\sotute|\\sot8te|\\sot9te|\\sotote|\\sotkte|\\sotjte"</td></tr>
<tr><td>"\\sotire|\\soti5e|\\soti6e|\\sotiye|\\sotige|\\sotife]"</td></tr>
</table>

Table S2. Performances for the single kernel CNN architectures. Network identifiers follow the structure b<n>-k<m>-f<l>-do<j>. Here, 'b<n>' denotes the batch size, with 'n' being the number of samples in each training batch. The segment 'k<m>' refers to the size of the convolutional kernel, expressed as a square with dimensions '<m>x<m>'. 'f<l>' specifies the number of filters in the convolutional layer, where 'l' is the quantity of filters used. Lastly, 'do<j>' indicates the dropout rate, with 'j' representing the percentage of dropout applied.

Single kernel	Kernel [size]	Batch [size]	Filters [#]	Dropout [%]	Accuracy (frozen) [epochs]	Accuracy (fine-tuned) [epochs]	Running time [min]
b8-k2-f128-do05	2	8	128	50	**98·00 [203]**	96·67 [48]	154·80
b8-k2-f128-do07	2	8	128	70	97·00 [114]	96·67 [3]	108·57
b8-k2-f256-do05	2	8	256	50	97·33 [73]	97·00 [1]	119·82
b8-k2-f256-do07	2	8	256	70	97·00 [48]	96·67 [1]	110·68
b16-k2-f128-do05	2	16	128	50	96·33 [23]	96·67 [121]	112·60
b16-k2-f128-do07	2	16	128	70	96·33 [92]	95·67 [1]	**87·15**
b16-k2-f256-do05	2	16	256	50	97·00 [39]	97·33 [24]	104·40
b16-k2-f256-do07	2	16	256	70	97·33 [69]	96·67 [1]	103·79
b8-k3-f128-do05	3	8	128	50	97·00 [37]	96·67 [1]	109·31
b8-k3-f128-do07	3	8	128	70	96·33 [140]	95·67 [1]	149·97
b8-k3-f256-do05	3	8	256	50	97·33 [53]	96·33 [1]	141·60
b8-k3-f256-do07	3	8	256	70	97·33 [111]	97·33 [1]	174·00
b16-k3-f128-do05	3	16	128	50	97·67 [128]	**97·67 [1]**	125·11
b16-k3-f128-do07	3	16	128	70	96·33 [83]	95·33 [1]	108·10

b16-k3-f256-do05	3	16	256	50	97·00 [30]	96·67 [69]	151·93
b16-k3-f256-do07	3	16	256	70	96·67 [47]	95·67 [20]	131·40

Table S3. Performances for the sequential kernel CNN architectures. Network identifiers are structured as b<n>-<k>x<m>-do<j>. In this format, 'b<n>' indicates the batch size, where 'n' is the number of samples in each training batch. The term '<k>x<m>' specifies a convolutional layer characterized by a square kernel of size '<k>x<k>' and using 'm' filters. For example, '2x128-3x256' describes a network composed of two sequential layers: the first with a 2x2 kernel and 128 filters, followed by the second with a 3x3 kernel and 256 filters. Finally, 'do<j>' represents the dropout rate, expressed as a percentage 'j%' of dropout applied.

Sequential kernels	Filters [#-#]	Batch [size]	Dropout [%]	Accuracy (frozen) [epochs]	Accuracy (fine-tuned) [epochs]	Running time [min]
b8-2x128-3x256-do05	128-256	8	50	97·67 [57]	98·00 [1]	119·67
b8-2x128-3x256-do07	128-256	8	70	97·33 [68]	96·67 [1]	131·40
b16-2x128-3x256-do05	128-256	16	50	97·33 [107]	97·00 [1]	119·09
b16-2x128-3x256-do07	128-256	16	70	97·00 [80]	97·00 [1]	**110·03**
b8-2x256-3x512-do05	256-512	8	50	97·67 [50]	97·67 [1]	172·20
b8-2x256-3x512-do07	256-512	8	70	97·67 [65]	**98·33 [2]**	183·00
b16-2x256-3x512-do05	256-512	16	50	**98·00 [19]**	97·67 [1]	140·40
b16-2x256-3x512-do07	256-512	16	70	97·33 [95]	97·33 [1]	184·80

Table S4. Performances for the parallel kernel CNN architectures. Network ids are coded as b<n>-(emb+2+3)x<m>-do<j>. Here, 'b<n>' specifies the batch size, with 'n' representing the number of samples in each training batch. The segment '(emb+2+3)x<m>' refers to a sequence of three layers: an initial embedding layer, followed by two parallel convolutional layers. The first of these convolutional layers utilizes a 2x2 kernel, while the second employs a 3x3 kernel, and both layers use 'm' filters. For example, '(emb+2+3)x128' indicates a network structure with an embedding layer followed by two parallel convolutional layers, the first with a 2x2 kernel and 128 filters, and the second with a 3x3 kernel and 128 filters. Finally, 'do<j>' denotes the dropout rate, where 'j' is the percentage of dropout applied.

**Parallel kernels**	**Filters [#]**	**Batch [size]**	**Dropout [%]**	**Accuracy (frozen) [epochs]**	**Accuracy (fine-tuned) [epochs]**	**Running time [min]**
b8-(emb+2+3)x128-do05	128	8	50	97·33 [71]	97·33 [1]	178·80
b8-(emb+2+3)x128-do07	128	8	70	97·33 [47]	97·33 [1]	**163·20**
b16-(emb+2+3)x128-do05	128	16	50	97·33 [46]	97·00 [1]	174·00
b16-(emb+2+3)x128-do07	128	16	70	97·33 [107]	97·33 [1]	212·40
b8-(emb+2+3)x256-do05	256	8	50	97·00 [7]	97·33 [18]	190·80
b8-(emb+2+3)x256-do07	256	8	70	**98·00 [82]**	97·33 [2]	232·80
b16-(emb+2+3)x256-do05	256	16	50	97·33 [48]	97·33 [1]	243·60
b16-(emb+2+3)x256-do07	256	16	70	97·67 [65]	**97·67 [1]**	257·40

Table S5. Performances for the deep-parallel kernel CNN architectures. Each network is identified using the code format b<n>-(emb+2+3)x<m>-(2+3)x<k>-do<j>. Here, 'b<n>' indicates the batch size with 'n' being the number of samples in each training batch. The term '(emb+2+3)x<m>' describes a sequence of three layers: an initial embedding layer, followed by two parallel convolutional layers. The first convolutional layer uses a 2x2 kernel and the second a 3x3 kernel, both employing 'm' filters. For instance, '(emb+2+3)x128' denotes a network with an embedding layer followed by two convolutional layers, one with a 2x2 kernel and 128 filters, and the other with a 3x3 kernel and 128 filters. This is succeeded by another pair of parallel layers, each with kernels of 2x2 and 3x3 sizes, respectively, and 'k' filters. Finally, 'do<j>' represents the dropout rate, where 'j' is the percentage of dropout applied.

Deep-parallel kernels	Filters [#-#]	Batch [size]	Dropout [%]	Accuracy (frozen) [epochs]	Accuracy (fine-tuned) [epochs]	Running time [min]
b8-(emb+2+3)x128-(2+3)x256-do05	128-256	8	50	**98 [30]**	**98 [11]**	**308·56**
b8-(emb+2+3)x128-(2+3)x256-do07	128-256	8	70	97·33 [92]	97·67 [1]	325·84
b16-(emb+2+3)x128-(2+3)x256-do05	128-256	16	50	97·67 [101]	**98·00 [17]**	379·12
b16-(emb+2+3)x128-(2+3)x256-do07	128-256	16	70	97·00 [70]	96·67 [2]	311·08
b8-(emb+2+3)x256-(2+3)x512-do05	256-512	8	50	**98·00 [133]**	97·67 [8]	786·6
b8-(emb+2+3)x256-(2+3)x512-do07	256-512	8	70	97·67 [98]	97·33 [1]	580·6
b16-(emb+2+3)x256-(2+3)x512-do05	256-512	16	50	96·67 [12]	97·00 [2]	423·04
b16-(emb+2+3)x256-(2+3)x512-do07	256-512	16	70	97·33 [45]	96·67 [1]	529·8
